# Geographic Spread of an AIDS-Resistant Mutation

**DOI:** 10.1371/journal.pbio.0030397

**Published:** 2005-10-18

The discovery in the 1990s of a gene variant that thwarts HIV infection triggered development of a promising new class of medications. The gene normally encodes a protein receptor, called CCR5, that sits on the surface of white blood cells. HIV gains entry to these cells through CCR5. The variant gene, or allele, contains a mutation—called Δ32, because 32 base pairs are deleted—that produces truncated CCR5 receptors that are useless to the virus, conferring resistance to individuals with both copies of the mutation, and delaying disease progression to those with one copy. The Δ32 mutation also raised interesting questions for evolutionary biologists.

About 10% of Europeans and inhabitants of western Asia carry the mutation, which researchers think evolved at least 700 years ago—yet HIV emerged only about 50 years ago. According to population genetics theory, for a mutation to be neutral, or confer no selective advantage, it would have to be much older to occur at such a high frequency in the population. This inconsistency raised the possibility that the mutation spread because it provided an advantage against some other selective factor, now thought to be smallpox.

The spread of advantageous alleles within a population is a fundamental aspect of evolution. While theoretical models have studied the dynamics of dispersal, few studies have tracked real-life examples or developed statistical methods to investigate the process. Now, John Novembre, Alison Galvani, and Montgomery Slatkin use the geographic distribution of the Δ32 mutation as an example of the process, and estimate the effects of selection relative to dispersal on Δ32 to shed light on its origins and spread through Europe. Their model shows that, given its age, the mutation spread rapidly due to long-range dispersal and intense selection to attain its current range.

The mutation follows a north–south spatial gradient, from northern Europe to Greece. Since the broadest area of high frequency is northeastern Europe—based on the population genetics assumption that a mutation originated where it is most abundant—one hypothesis is a Viking origin. The Viking hypothesis, first put forth in the 1990s, proposes that the allele was present in Scandinavia at least 1,000–1,200 years ago, and Vikings carried it north to Iceland, east to Russia, and south to central and southern Europe. Alternately, the mutation may have arisen in central Europe and then increased in frequency in the north (if selection pressures there increased the advantage of having the allele), or it may have arisen in the north and spread as individuals (not Vikings) migrated to other areas. [Fig pbio-0030397-g001]

**Figure pbio-0030397-g001:**
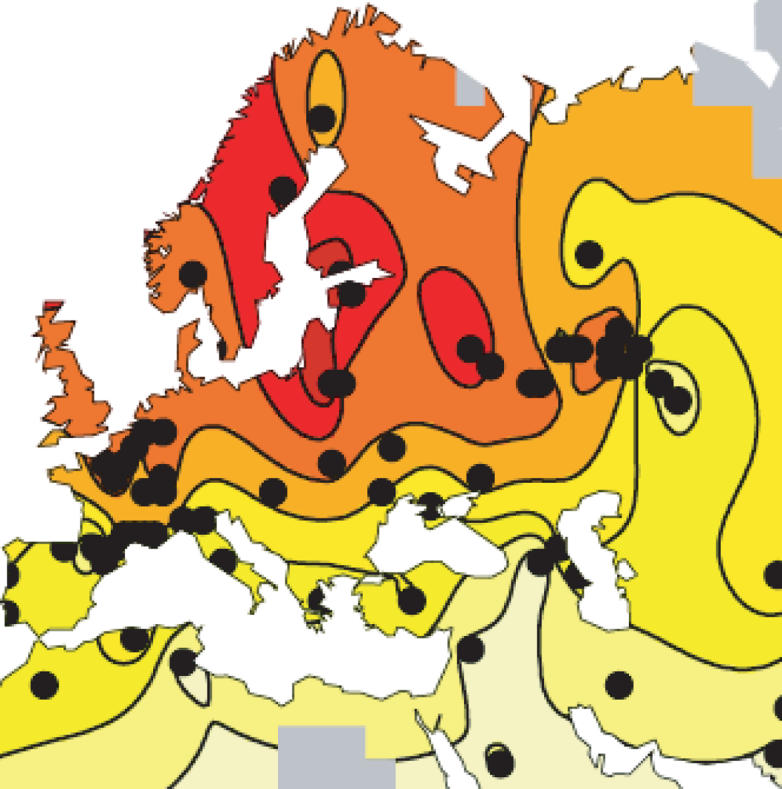
This contour map shows the European distribution of the CCR5 Δ32 variant, which was used to develop a method for studying the geographic spread of gene variants under positive selection

To quantitatively test these competing hypotheses, Novembre et al. used allele frequencies collected from 71 locations and simulated the spread of the mutation by varying the origin of the mutation, the relative strength of selection to dispersal, and the strength of gradients in selection. The authors then calculated the probability of the allele frequency data given the parameters, and then found the parameter set that best fit the data. The results indicate that, given its age, strong selection and long-range dispersal (perhaps through trade routes or migrations) are the most likely explanation for the present distribution of the Δ32 mutation. The data show a north–south gradient in allele frequency, supporting a modest north–south gradient in selection intensity. As for the mutation's provenance, a northern European origin is supported only by assuming uniform selection; assuming a selection gradient points to a more southern origin, perhaps in Spain, or in northern Germany (when data from Iceland are included).

Altogether, the results suggest caution regarding the Viking hypothesis: though the long-range dispersal findings are consistent with the hypothesis, the authors conclude, a southern origin “raises the possibility that the allele arose outside of Scandinavia and spread into the region via dispersers from the south.” The results also show that modest selection gradients—where the intensity of selection differs slightly between distant locations—allow for an allele to originate far from its current abundance.

By applying a standard model of the spatial spread of an advantageous allele to a map of Europe and west Asia and adding a statistical component to the model, Novembre et al. could analyze the allele frequency data from these two regions. Though a new study by Pardis Sabeti et al. (DOI: 10.1371/journal.pbio.0030378), also in this issue, presents evidence that CCR5 Δ32 is older than previously estimated and that its frequency could be explained by neutral selection, the findings don't discount the validity of the model presented here. In fact, if selection on CCR5 Δ32 were weaker, then the dispersal estimated in this model would become weaker—falling more in line with historical estimates. However the mutation arose, this approach will allow researchers to tease apart the relative contributions of dispersal patterns and local selection pressures to study the geographic distribution and evolutionary history of any mutation—critical tools for tracing the relatively recent evolutionary history of humans. —*Liza Gross*

